# The Validity and Reliability of Automatic Tooth Segmentation Generated Using Artificial Intelligence

**DOI:** 10.1155/2023/5933003

**Published:** 2023-07-18

**Authors:** Ammar Sh. Al-Ubaydi, Dheaa Al-Groosh

**Affiliations:** ^1^College of Dentistry, University of Baghdad, Baghdad, Iraq; ^2^Ministry of Health, Baghdad, Iraq; ^3^Orthodontic Department, College of Dentistry, University of Baghdad, Baghdad, Iraq

## Abstract

This study aimed at evaluating the precision of the segmented tooth model (STM) that was produced by the artificial intelligence (AI) program (CephX®) with an intraoral scan (IOS) and insignia outcomes. *Methods*. 10 patients with Cl I malocclusion (mild-to-moderate crowding) who underwent nonextraction orthodontic therapy with the Insignia™ system had IOS and CBCT scans taken before treatment. AI was used to produce a total of 280 STMs; each tooth will be measured from three aspects (apexo-occlusal, mesiodistal, and labiolingual) for DICOM and STL formats. Also, root volume measurements for each tooth generated by using the CephX® software and Insignia™ system were compared. The software used for these measurements was the OnDemand3D program used for the multiplanar reconstruction for DICOM format and Geomagic® Control X™ used for STL format. *Statistics*. An intraclass correlation (ICC) analysis was used to check the agreement between the volume measurement of the segmented teeth generated by using the CephX® and Insignia™ system. Also, it was used to check the agreement between the STL (IOS), STL (CephX®), and DICOM tooth models. In addition, it was used to determine the intraexaminer repeatability by remeasuring five randomly selected individuals two weeks after the initial measurement. After confirmation of the data normality using the Shapiro–Wilk test, the right and left tooth models and the differences between the DICOM, CephX® (STL), and IOS (STL) tooth models were compared using a paired *t*-test. The STL (IOS), STL (CephX®), and DICOM tooth models were compared utilizing the ANOVA test. *p* < 0.05 was set as the statistical significance level. *Result*. Overall data showed good agreement with ICC. The measurements of the various tooth types on the right and left sides did not differ significantly. Also, there was no significant difference between the three groups. *Conclusions*. The automatic AI approach (CephX®) may be advised in the clinical practice for patients with mild crowding and no teeth restorations due to its speed and effectiveness.

## 1. Introduction

In medicine, dentistry, and orthodontics, imaging is used to show the patient's “anatomic reality,” which in turn brings the best fit in diagnosis and a proper treatment strategy, ultimately resulting in successful treatment outcomes [1]. For years, in orthodontics, a patient's three-dimensional (3D) status was assessed using two-dimensional radiographs such as orthopantomography and cephalometric X-ray [2]. However, using two-dimensional X-ray projection to examine a three-dimensional object has several well-known problems, including geometric distortion, superimposition of neighboring structures, magnification, projective displacement, linear projective transformation, and rotational inaccuracies [3–5].

Cone-beam computed tomography (CBCT), first used in dentistry in 1998, has completely changed how diagnosis could be made for dentistry and orthodontics, particularly by delivering precise and high-resolution three-dimensional (3D) imaging from small and affordable devices. Furthermore, compared with traditional CT, the patient receives much less radiation exposure [6–8].

The operating CBCT software, DICOM (Digital Image and Communication in Medicine) file, is used to store and transmit the CBCT's 3D medical imaging data [9]. The DICOM file can produce a 3D rotating image, including crowns, roots, and bone, which is segmented so that teeth can be covered or removed to show underlying structures. Furthermore, it can exhibit a series of two-dimensional slices in three-dimensional planes [10].

Recognizing a root from a bone is a complicated process, and it is difficult to identify the entire length and anatomy of the roots, even with the necessary expertise, since the two structures have almost the same density. However, many software tools may help in teeth segmentation but require considerable operator experience, effort, and time in addition to validity checks [10–12].

To generate a 3D user-friendly landscape, the DICOM file should be converted to STL (Standard Triangle Language) format to convert and segment the teeth and jaws. These files are used for printing 3D objects in the medical and industrial fields. There are various ways to create an STL model, including manual segmentation using a Materialize Interactive Medical Image Control System (MIMICS) program or automatic image segmentation using an artificial intelligence (AI) program such as CephX® (Orca Dental Al, Las Vegas, NV) [13], U-Net, residual U-Net, and Xception U-Net architectures [14]. There was a consensus on the precision of the manual method; however, it required a long time to obtain the 3D segmented tooth [10]. On the other hand, the advancement in artificial intelligence has allowed it to become an integral component of many medical applications, such as in DLAD (deep learning-based automatic detection) to analyze chest radiographs and detect abnormal cell growth [15]. It was also used in LYmph Node Assistant (LYNA), which analyzed histology slide-stained tissue samples to identify metastatic breast cancer tumors from lymph node biopsies [16]. In dental radiology, artificial intelligence is a reliable tool that segments and recognizes complex imaging data and can automatically convert a DICOM file obtained from a CBCT scan into a segmented STL image within a few minutes [17].

Recent years have seen the utilization of 3D computer models to create setups and enable indirect bracket positioning for customized orthodontic devices. For example, the Insignia™ system (Trademark of Ormco Corporation, Orange, CA; https://www.ormco.com) integrated the CBCT-generated TruRoot® data for precise root positions and low-dose radiation exposure, which provides more reliable Insignia-simulated treatment results [18].

This study was designed to assess the validity and reliability of a 3D tooth model created using a deep learning technique program (CephX®) with its 2D slices of the CBCT image first and then to compare this segmented model to the 3D tooth model generated by the intraoral scan and the Insignia™ system logarithms.

## 2. Materials and Methods

This study, a part of a prospective ongoing clinical trial registered in ClinicalTrials.gov (NCT05549089), was approved by the research and ethics board committee (no. 624422/2021). The orthodontic records, including CBCT and intraoral scan images, intraoral and extraoral photos, a 3D-printed virtual setup model, and an initial plaster model of ten participants, were selected at the orthodontics department, College of Dentistry/University of Baghdad and from the private clinic.

### 2.1. The Study Sample

The participants included sixteen patients, with only 10 patients fulfilling the inclusion criteria of 15–30 years of age with class I malocclusion, a full set of permanent teeth, and mild-to-moderate crowding (irregularity index < 6 mm) [19] who required a nonextraction orthodontic treatment with the fixed appliance (Insignia™ system), while six patients were excluded according to the exclusion criteria which included patients with large fillings, malformed tooth shape, significant restorative or prosthodontic therapy, dilacerated roots, or severe root resorptions. Post hoc power analysis was performed using G^*∗*^Power (version 3.1.9.4, Win) [20] for one-way ANOVA tests assuming *α* = 0.05 and a power of 0.80. Based on this assumption, a sensitivity analysis was carried out based on the anticipated sample size (*N* = 10 patients = 280 tooth model) resulting in a minimum detectable effect size of Cohen's *d* = 0.599.

### 2.2. Methodology

**Figure 1:**
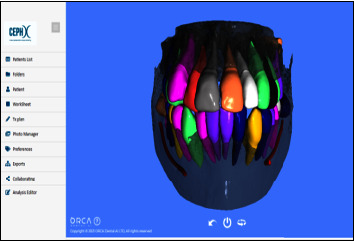
A segmented 3D model of the teeth and jaws produced by CephX® web viewer.

A pretreatment intraoral scan (IOS) and a CBCT scan were performed for each patient using a 3D optical laser scanner (3Shape TRIOS 3, Copenhagen, Denmark) and a SOREDEX® (Tuusula, Uusimaa, Finland) CBCT machine for 3D digital analysis. To obtain CBCT images, the following protocols and specifications were used for an appropriate field of vision (FOV): the working regime was set at a field of view of 200 × 179 mm^2^, 80 kV, 5 mA, and a voxel size of 0.39 mm [10]. The CBCT slices obtained by using the CRANEX® software (version 2.1.0.30) were exported in DICOM format and stored on recordable media. The CephX® web viewer (Orca Dental Al, Las Vegas, NV) received the DICOM file and converted it into an STL format automatically with a segmented 3D representation of the teeth and jaws, as shown in Figure 1. This study includes two parts: in the first part of the study, linear measurements of teeth from three aspects (apexoocclusal, mesiodistal, and labiolingual) in both the DICOM and STL formats were performed. Two software programs used for these measurements include OnDemand3D program version 1.0.10.5385 (Cybermed Inc., Seoul, Korea) for the multiplanar reconstruction of DICOM format and Geomagic® Control X™ software (version 2020.1.1; 3D Systems Inc., Rock Hill, SC, USA) for STL format. In the second part of the study, the volume measurement of 3D teeth models that were generated by using the CephX® web viewer and those exported from the Insignia™ system using Geomagic® Control X™ (CX) software (version 2020.1.1; 3D Systems Inc., Rock Hill, SC, USA) was performed.

### 2.3. Linear Measurements

(1) The apexoocclusal measurements of the teeth include the following:
  - Anterior teeth: from the incisal edge's midpoint to the root apex
  - Premolars: from the buccal root apex to the buccal cusp tip
  - Upper molars: from the palatal root apex to the mesiopalatal cusp tip
  - Lower molars: from the mesial root apex to the mesiobuccal cusp tip
(2) The mesiodistal width of the teeth was measured 3 mm gingivally away from the incisal edge of the anterior teeth and the buccal cusp tip of the posterior teeth
(3) The labiolingual width of the teeth was measured from the midpoint of the mesiodistal width for both labial and lingual sides.

#### 2.3.1. Measurements of the Teeth in DICOM Format

**Figure 2:**
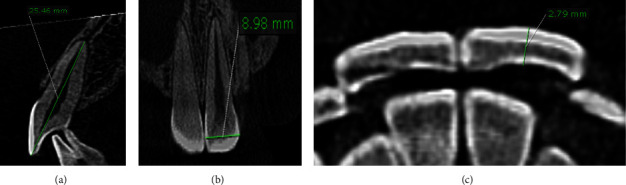
Tooth measurements from three aspects of DICOM images: (a) apexoocclusal, (b) mesiodistal, and (c) labiolingual aspects.

The measurements of the teeth in the DICOM format were performed in the following sequence (Figure 2):

1. The apexoocclusal length was measured from the sagittal view
2. The mesiodistal width of the teeth was measured from the coronal view, 3 mm gingivally away from the incisal edge and buccal cusp tips
3. The labiolingual width was measured from the axial view at the midpoint of the previous mesiodistal width

#### 2.3.2. Measurements of the Teeth in STL Format

**Figure 3:**
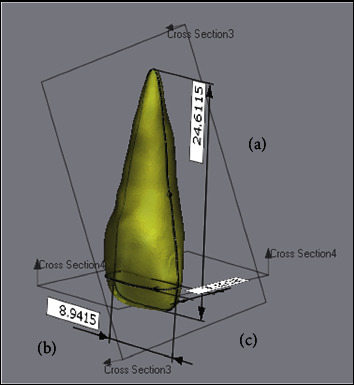
Tooth measurements from three aspects using STL images of CephX®: (A) apexoocclusal, (B) mesiodistal, and (C) labiolingual.

**Figure 4:**
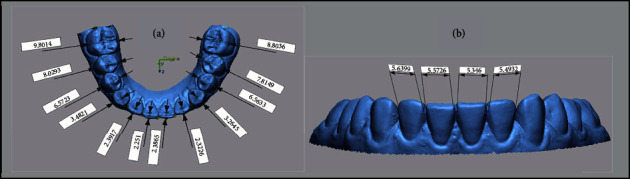
Tooth measurements from two aspects using STL images of the intraoral scanner: (a) labiolingual and (b) mesiodistal.

The measurements of the teeth in the STL format were performed after the superimposition of CBCT images and IOS (Figures 3 and 4):

1. The apexoocclusal length was measured from the sagittal aspect. This measurement was performed for the STL image that was produced from the CBCT image.
2. The mesiodistal width was measured from the coronal aspect, 3 mm gingivally away from the incisal edge and buccal cusp tips in the axial plane.
3. The labiolingual width was measured from the axial aspect at the midpoint of the previous mesiodistal width.

### 2.4. Volume Measurement

In the other part of the study, the volume measurement of 3D teeth models that were generated by using the CephX® web viewer and those exported from the Insignia™ system was performed. The exported 3D teeth models from the Insignia™ system were created by merging the clinical crown from the intraoral scan and the segmented root from the CBCT image; therefore, the coronal portion should be excluded from the measurement.

To standardize the measurement, the crown volume from the IOS was subtracted from the volume of segmented teeth by using the CephX® software and Insignia™ system.

### 2.5. Root Volume Assessment

**Figure 5:**
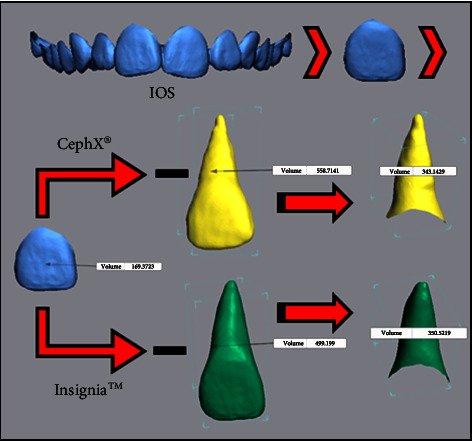
Root volume measurement of a segmented tooth. It was obtained by subtracting the volume measurement of the segmented teeth generated by using the CephX® software and Insignia™ system from the IOS clinical crown volume measurement.

The crown volume from the IOS and root volume of each segmented tooth generated by using the CephX® software and Insignia™ system was calculated using Geomagic® Control X™ (CX) (3D Systems, USA).  Figure 5 shows the sequences of volume measurement as follows:

1. The IOS dental model was imported into the Geomagic® Control X™ (CX) software, and then, the gingival region was trimmed to the cervical area of the clinical crowns
2. Then, each tooth model was imported into the CX software by using the CephX® software and Insignia™ system
3. Afterwards, each clinical crown volume and the segmented tooth models were measured by selecting the measure volume option, followed by the enclosed volume function
4. Finally, the IOS clinical crown volume measurement was subtracted from the volume measurement of both segmented models to achieve the root volume for each segmented tooth.

### 2.6. Statistical Analysis

Statistical Package for the Social Sciences (SPSS) was used to conduct the statistical analysis (version 26.0; IBM SPSS, Armonk, NY). The means and standard deviations of the measurements in the DICOM, STL (CephX®), and STL (IOS) tooth formats were calculated. An intraclass correlation (ICC) analysis was used to check the agreement between the volume measurement of the segmented teeth generated by using the CephX® web viewer and Insignia™ system. Also, it was used to check the agreement between the DICOM, STL (CephX®), and STL (IOS) tooth models. In addition, it was used to determine intraexaminer repeatability, which showed high agreement when 60 teeth were randomly selected and remeasured after two weeks from the initial measurement. After confirmation of the data normality using the Shapiro–Wilk test, the difference between the right and left teeth in the DICOM, STL (CephX®), and STL (IOS) formats was compared using a paired-sample *t*-test. The DICOM, STL (CephX®), and STL (IOS) tooth models were compared utilizing the one-way ANOVA test with a large effect size of 0.18; *p* value <0.05 was set as the statistical significance level.

## 3. Results

The results showed that, for the 3D tooth model produced by using the CephX® web viewer in STL formats, the average of the apexoocclusal length between the left and right sides for the maxillary and mandibular teeth (a mean difference of 0.43 mm and 0.11 mm, respectively) had no significant difference (Table 1). In addition, Tables 2 and 3 reveal that there was no significant difference between the left and right sides for the mesiodistal width of the maxillary and mandibular teeth (a mean difference of 0.06 mm and 0.07 mm, respectively) and for the labiolingual widths of the maxillary and mandibular teeth (a mean difference of 0.01 mm and 0.10 mm, respectively).

Regarding the DICOM formats of tooth dimensions, the apexoocclusal length of the maxillary and mandibular teeth (a mean difference of 0.40 mm and 0.29 mm, respectively) had no significant difference (Table 1). Moreover, Tables 2 and 3 show a nonsignificant difference between the right and left sides for the mesiodistal and labiolingual widths of the maxillary and mandibular teeth (a mean difference of 0.05 mm, 0.08 mm, and 0.06 mm, 0.04 mm, respectively).

On the other hand, Table 4 compares the DICOM format and the STL (CephX) format and finds that the apexoocclusal lengths of the maxillary and mandibular teeth (a mean difference of 0.18 mm and 0.20 mm, respectively) had a nonsignificant difference between the two groups. Similarly, Tables 5 and 6 compare the mesiodistal and labiolingual widths between the DICOM, STL (CephX), and STL (IOS) formats, revealing that the mesiodistal width of the maxillary and mandibular teeth (a mean difference 0.17 mm for both arches) and the labiolingual widths of the maxillary and mandibular teeth (a mean difference 0.03 mm and 0.17 mm, respectively) had no significant difference between the three groups.

The agreement between the STL (IOS) and STL (CephX) tooth models was higher than that between the STL (IOS)-DICOM tooth models and STL (CephX)-DICOM tooth models in mesiodistal and labiolingual dimensions (Tables 5 and 6).

There was good agreement between the root volume measurement generated by using the CephX® and Insignia™ system (0.881), as shown in Table 7.

## 4. Discussion

Artificial intelligence technology has allowed the development of innovative diagnostic and treatment systems for radiology, imaging technology, ultrasonography, and pathology. Thus, this can significantly raise the standard and effectiveness of clinical practice. In addition, technology is gradually altering the conventional medical model, setting a course and trend for future advancements in human medicine [21]. Deep learning technology has improved artificial intelligence, enabling it to assess data like humans, recognize data in text, image, and voice formats, and perform image categorization, segmentation, and enhancement. The CephX® tooth-modelling service was used to implement the automatic deep learning method employing convolutional neural network characteristics [10].

The examiners' experience may impact the reproducibility of the measuring process for various formats of tooth models. Therefore, to reduce reproducibility errors in this study, a single researcher with expertise in the topic carried out the entire investigation.

The current investigation measured the pretreatment CBCT-scanned teeth using 3D reverse engineering software (Geomagic® Control X™, 3D Systems, USA). Dental Monitoring and Rapidform 3D software are options for already accessible software. Geomagic software is frequently used to create digital 3D models and CAD assemblies and primarily offers the ability to handle STL or CAD file formats. According to a study on the reliability of the Geomagic® Control X™ program, the 3D digital dental models created by it were precise enough to be applied in clinical settings [22].

In this study, there was a high correlation between the DICOM format and segmented STL format (CephX®), which is in agreement with the findings of Tarazona et al. [23], who compared twenty-seven models created by segmenting CBCT with the same twenty-seven models obtained by a digital method and found strong intraexaminer repeatability and a high correlation between the two approaches. However, segmented 3D models from CBCT images tend to underestimate tooth sizes, although this has no clinical ramifications when we perform calculations such as the discrepancy index or Bolton index.

Kau et al. [24] and Paredes et al. [25] compared the 3D CBCT segmented method to other digital processes and found that the 3D CBCT segmented method underestimated the size of the teeth, which is similar to our study that found subestimation of the mesiodistal width of the 3D segmented tooth model produced from CBCT images in the lower teeth because it is hard for CBCT technology to capture anatomic contact points [18]. Wiranto et al. [26] found the same finding but in the upper teeth.

Also, our study found good agreement between the CephX® (STL) image and the IOS (STL) image in mesiodistal and labiolingual dimensions but higher in the labiolingual dimension. This result may be due to the free lingual and buccal surfaces; therefore, detecting points on them is more accessible and precise than mesiodistal measurement.

Also, our results matched those of Flügge et al. [27], who found no difference between the 3D CBCT-segmented and 3D scanner models. Finally, a few articles in the scientific literature look at the dental size of nonsegmented 3D CBCT. One possible reason is confusion when finding the right-view slices (axial, coronal, and sagittal) to measure teeth sizes.

Unlike the current study, Wang et al. [28] found statistically significant differences between the reference and test models in vivo buccolingual, mesiodistal, and root length measurements. They hypothesize that the discrepancy may be attributable to variances in the scanning precision of the CBCT systems employed.

The results of the current study agreed with those of Sang et al. [29] who discovered that the reconstructed 3D tooth model from CBCT data could obtain a high linear, volumetric, and geometric accuracy. It is possible that the nonsignificant difference in this study is because a more accurate segmentation technique was applied through the use of deep learning-based automatic detection.

A crucial stage in creating an individual tooth model is teeth segmentation, and precise segmentation is essential. Several software applications for automatic segmentation have been made available in dentistry. Therefore, a technique that isolates the tooth, including the root, from the alveolar bone in CBCT images without removing the alveolar bone is preferred. This study's software is typically used for 3D modelling and analyzing medical images. The teeth segmentation from the alveolar bone proved challenging, unlike the medical segmentation of other anatomical structures such as the heart or pelvic bone. Essentially, the software carries out segmentation by discriminating and taking various degrees of several anatomic structures [17].

According to Lee et al. [10], manual tooth segmentation using the Mimics software required fifteen minutes per tooth and six hours for twenty-four teeth, including the first molar. One minute was required to segment each tooth using an automatic method, and it took twenty-four minutes to segment all of them including the first molar.

A systematic review and meta-analysis [30] suggested that digital 3D models generated from CBCT are reproducible for all measurements when intraexaminer assessment is considered, supporting the result of this study.

Since CBCT imaging was introduced to orthodontics, a volumetric analysis has become increasingly commonplace for anatomical visualization and biomechanical purposes [31]. Because of recent developments in CBCT technology, it is now possible for this segmentation method to become the standard of care in orthodontic practices. However, to reach its full potential in everyday diagnostics and treatment planning, it must first be validated by studies examining its accuracy and reliability [32]. Numerous research studies have concentrated their attention on the feasibility of using CBCT as a method for determining root lengths as well as root volumes [27–47].

Several studies have used CBCT to assess teeth in real life by isolating individual teeth from their surrounding context [31, 48–50]. Li et al. [34] found no statistically significant difference between in vitro and in vivo CBCT tooth volume assessments.

The current study compared the volume measurements of segmented teeth from CBCT images using two distinct programs, which were the CephX® software and Insignia™ system. As can be seen from the data presented here, neither the CephX® nor the Insignia™ groups differed significantly from one another. Based on these results, it appears that both programs are trustworthy for use in in vivo experiments. Since all varieties of 3D software are utilized to determine a volume generated from CBCT, it would appear that alterations of software or CBCT equipment have no substantial clinical significance when voxel sizes remain consistent [37]. However, the means of root volumes generated by using the Insignia™ system were always slightly smaller than those generated by using CephX®, and this may be due to the smoothening process of the segmented root surface [29].

The clinical importance of using artificial intelligence system that utilizes deep learning with proper models can successfully improve diagnosis and treatment plan and assessment of multiple aspects during orthodontic treatment. This technique can be used for the first step of a fully automated orthodontic diagnostic system in the future. It is necessary to assess future research employing a big sample size and another AI programme for the examination of dental models.

## 5. Conclusion

Compared to CBCT and IOS images, the automatic segmentation method demonstrated comparable accuracy in three-dimensional aspects. The assessment of in vivo tooth volume measurement between two different 3D imaging software programs (CephX® and Insignia™) was reliable. Based on this research, automatic AI may be advised for patients with mild crowding and no teeth restorations. Further work is required to confirm whether the presence of restorations and moderate/severe crowding will affect the AI result.

## Figures and Tables

**Table 1:** Difference between L and R sides for the upper and lower teeth in the apexoocclusal aspect.

Apexoocclusal										
Tooth no.	CephX				Sig.	DICOM				Sig.
	R		L			R		L		
	Mean	SD	Mean	SD		Mean	SD	Mean	SD	
*Maxilla*										
1	22.69	2.03	22.29	2.16	0.112	23.69	1.48	23.32	2.10	0.482
2	21.48	1.38	20.91	1.67	0.233	22.27	1.22	21.79	1.68	0.468
3	25.74	2.52	24.79	1.97	0.091	26.56	2.44	25.87	2.01	0.084
4	20.55	1.64	20.66	1.38	0.810	21.73	1.33	21.23	1.68	0.391
5	20.58	1.77	19.78	1.44	0.104	21.33	1.67	20.83	1.18	0.233
6	20.37	0.71	20.39	0.47	0.892	21.06	0.93	21.26	1.06	0.691
7	19.94	1.00	19.53	1.18	0.069	20.65	0.76	20.20	1.24	0.493

*Mandible*										
1	20.69	1.15	19.69	1.76	0.079	21.43	1.03	20.86	1.92	0.349
2	21.33	1.20	21.57	2.06	0.572	22.66	1.29	22.21	1.43	0.366
3	24.85	1.91	24.97	1.41	0.903	26.11	1.77	25.44	1.31	0.252
4	21.35	1.64	21.60	1.71	0.621	22.29	1.18	23.00	2.23	0.397
5	21.74	1.85	21.27	1.72	0.059	22.11	1.13	21.77	1.83	0.493
6	20.79	2.06	21.07	2.59	0.581	21.54	2.04	21.30	2.13	0.143
7	20.40	2.53	20.18	2.00	0.634	21.10	2.58	20.65	2.45	0.428

**Table 2:** Difference between L and R sides for the upper and lower teeth in the mesiodistal aspect.

Mesiodistal															
Tooth no.	CephX				Sig.	DICOM				Sig.	IOS				Sig.
	R		L			R		L			R		L		
	Mean	SD	Mean	SD		Mean	SD	Mean	SD		Mean	SD	Mean	SD	
*Maxilla*															
1	8.62	0.34	8.94	0.51	0.113	8.48	0.36	8.60	0.55	0.363	8.69	0.71	8.69	0.77	0.972
2	6.92	0.60	6.64	0.48	0.432	6.63	0.62	6.36	0.24	0.281	6.73	0.33	6.66	0.54	0.747
3	7.76	0.48	7.67	0.68	0.588	7.71	0.11	7.65	0.54	0.783	7.77	0.84	7.93	0.79	0.371
4	7.31	0.55	7.26	0.53	0.857	7.10	0.55	7.03	0.65	0.866	7.21	0.77	7.31	0.55	0.584
5	6.43	0.59	6.89	0.52	0.372	6.86	0.69	6.44	0.40	0.421	6.54	0.41	6.55	0.14	0.938
6	10.54	0.96	10.46	0.77	0.681	10.40	0.71	10.55	0.47	0.637	10.33	0.59	10.63	1.05	0.359
7	10.02	0.58	10.14	0.43	0.549	9.62	0.52	9.82	0.43	0.511	10.09	0.77	10.49	0.75	0.329

*Mandible*															
1	4.91	0.40	4.95	0.39	0.643	5.36	0.28	5.23	0.30	0.173	5.38	0.43	5.37	0.57	0.971
2	5.57	0.47	5.47	0.60	0.525	5.81	0.29	5.61	0.28	0.168	5.88	0.37	5.92	0.34	0.394
3	6.94	0.65	7.00	0.77	0.778	6.82	0.54	6.85	0.75	0.844	7.08	0.70	6.88	0.62	0.087
4	6.77	0.44	6.78	0.81	0.963	6.72	0.65	7.06	0.63	0.116	7.15	0.51	7.18	0.54	0.822
5	7.15	0.40	7.05	0.28	0.176	7.09	0.78	7.18	0.56	0.781	7.08	0.39	7.18	0.71	0.716
6	10.79	1.14	10.84	1.27	0.891	11.14	0.91	11.33	0.81	0.535	11.13	0.60	10.91	0.99	0.671
7	10.51	1.01	11.03	0.67	0.087	11.07	0.66	11.30	0.58	0.088	10.33	0.98	10.39	1.14	0.843

**Table 3:** Difference between L and R sides for the upper and lower teeth in the labiolingual aspect.

Labiolingual															
Tooth no.	CephX				Sig.	DICOM				Sig.	IOS				Sig.
	R		L			R		L			R		L		
	Mean	SD	Mean	SD		Mean	SD	Mean	SD		Mean	SD	Mean	SD	
*Maxilla*															
1	3.40	0.53	3.49	0.42	0.739	3.33	0.29	3.41	0.47	0.521	2.77	0.40	2.95	0.39	0.286
2	3.07	0.44	3.16	0.30	0.577	3.12	0.29	3.04	0.23	0.548	2.72	0.45	2.68	0.27	0.648
3	4.70	0.68	4.85	0.74	0.658	4.92	0.61	4.06	2.37	0.437	3.91	0.67	4.61	1.94	0.323
4	9.76	0.79	9.32	0.49	0.083	9.41	0.73	9.37	0.47	0.877	8.80	0.78	8.66	0.66	0.354
5	9.42	0.58	9.34	0.28	0.792	9.35	0.07	9.42	0.27	0.485	8.61	0.32	8.72	0.24	0.287
6	11.52	0.83	11.32	0.76	0.222	11.71	0.43	11.01	0.61	0.068	9.66	0.55	9.79	0.60	0.591
7	11.19	0.84	10.84	0.36	0.196	11.30	0.54	11.43	0.26	0.611	9.58	1.02	9.57	0.76	0.959

*Mandible*															
1	2.61	0.50	2.81	0.36	0.216	2.65	0.53	2.91	0.50	0.133	2.19	0.39	2.45	0.52	0.068
2	2.63	0.15	2.64	0.27	0.867	2.79	0.51	2.73	0.37	0.688	2.29	0.30	2.37	0.20	0.257
3	4.42	0.66	4.37	0.99	0.897	4.59	0.48	4.28	0.55	0.351	3.26	0.43	3.32	0.37	0.452
4	7.33	0.27	7.20	0.52	0.576	7.63	0.37	7.71	0.33	0.769	6.89	0.53	6.78	0.26	0.433
5	8.31	0.45	8.14	0.38	0.586	8.33	0.44	8.47	0.40	0.667	7.45	0.36	7.69	0.66	0.395
6	10.08	0.70	10.30	0.77	0.543	10.39	0.33	10.41	0.39	0.875	8.95	0.66	9.20	0.44	0.544
7	10.00	0.67	10.04	0.75	0.863	10.11	0.41	10.23	0.45	0.104	8.79	0.77	8.81	0.95	0.919

**Table 4:** Difference between CephX and DICOM for the upper and lower teeth in the apexoocclusal aspect.

Apexoocclusal										
Tooth no.	CephX				DICOM				Sig.	ICC
	Mean	SD	Max	Min	Mean	SD	Max	Min		CephX-DICOM
*Maxilla*										
1	22.49	2.08	24.36	18.98	22.22	2.43	26.18	19.57	0.834	0.999
2	21.20	1.46	23.27	19.52	21.32	3.41	27.25	18.55	0.918	0.998
3	25.26	2.21	27.64	22.22	21.63	2.90	26.57	19.39	0.063	0.996
4	20.6	1.43	22.13	18.41	21.68	2.59	24.45	17.96	0.541	0.993
5	20.18	1.57	21.30	17.66	21.14	1.62	23.51	19.41	0.528	0.994
6	20.38	0.58	21.15	19.68	22.04	1.44	23.58	20.29	0.054	0.962
7	19.74	1.08	21.50	18.82	21.12	1.32	22.52	19.55	0.093	0.804

*Mandible*										
1	20.33	1.15	21.41	18.77	20.27	1.01	21.36	19.06	0.736	0.972
2	21.45	1.63	23.05	19.35	21.60	1.39	22.65	19.56	0.499	0.978
3	24.91	1.37	26.14	22.87	24.97	1.48	26.34	22.85	0.493	0.998
4	21.48	1.59	23.35	19.23	21.65	1.71	23.99	19.31	0.314	0.991
5	21.51	1.78	23.29	19.02	21.94	1.43	23.96	20.54	0.248	0.943
6	20.93	2.28	24.29	18.80	21.02	2.47	24.32	18.51	0.663	0.993
7	20.29	2.23	23.32	17.90	20.88	2.45	23.87	18.88	0.093	0.966

Total agreement (ICC)										0.971

**Table 5:** Difference between CephX, DICOM, and IOS for the upper and lower teeth in the mesiodistal aspect.

Mesiodistal										
Tooth no.	CephX		DICOM		IOS		Sig.	ICC		
	Mean	SD	Mean	SD	Mean	SD		CephX-DICOM	CephX-IOS	DICOM-IOS
*Maxilla*										
1	8.78	0.40	8.54	0.45	8.69	0.73	0.793	0.839	0.842	0.836
2	6.78	0.41	6.50	0.40	6.69	0.39	0.538	0.457	0.691	0.848
3	7.71	0.57	7.68	0.31	7.85	0.80	0.897	0.853	0.907	0.639
4	7.28	0.47	7.07	0.40	7.26	0.65	0.784	0.896	0.812	0.871
5	6.66	0.24	6.66	0.22	6.55	0.27	0.712	0.866	0.534	0.718
6	10.50	0.85	10.48	0.51	10.48	0.79	0.998	0.658	0.919	0.755
7	10.08	0.46	9.72	0.37	10.29	0.65	0.243	0.585	0.838	0.546

*Mandible*										
1	4.93	0.38	5.30	0.28	5.37	0.48	0.198	0.352	0.703	0.659
2	5.52	0.51	5.71	0.25	5.90	0.35	0.333	0.664	0.759	0.663
3	6.97	0.68	6.84	0.64	6.98	0.66	0.931	0.887	0.971	0.951
4	6.77	0.59	6.89	0.61	7.16	0.50	0.559	0.938	0.844	0.809
5	7.10	0.34	7.14	0.61	7.13	0.50	0.989	0.555	0.848	0.917
6	10.82	1.15	11.24	0.80	11.02	0.62	0.757	0.896	0.783	0.926
7	10.77	0.82	11.19	0.61	10.36	1.01	0.241	0.937	0.691	0.655

Total agreement (ICC)								0.741	0.796	0.771

**Table 6:** Difference between CephX, DICOM, and IOS for the upper and lower teeth in the labiolingual aspect.

Labiolingual											
Tooth no.	CephX		DICOM			IOS		Sig.	ICC		
	Mean	SD	Mean		SD	Mean	SD		CephX -DICOM	CephX -IOS	DICOM-IOS
*Maxilla*											
1	3.45	0.39	3.37	0.37		3.51	0.32	0.831	0.818	0.822	0.598
2	3.12	0.33	3.08	0.23		3.05	0.39	0.588	0.885	0.891	0.923
3	4.77	0.6	4.97	0.65		4.81	0.6	0.856	0.953	0.957	0.961
4	9.54	0.63	9.40	0.56		9.59	0.77	0.889	0.851	0.858	0.804
5	9.39	0.33	9.38	0.16		9.37	0.43	0.683	0.866	0.870	0.848
6	11.42	0.78	11.37	0.42		11.6	0.56	0.822	0.767	0.771	0.566
7	11.02	0.60	11.37	0.34		10.9	0.60	0.373	0.671	0.677	0.629

*Mandible*											
1	2.71	0.41	2.78	0.49		2.64	0.54	0.895	0.952	0.953	0.935
2	2.63	0.21	2.76	0.41		2.46	0.20	0.288	0.828	0.829	0.679
3	4.40	0.72	4.44	0.40		4.13	0.95	0.767	0.869	0.876	0.533
4	7.27	0.33	7.67	0.16		6.95	1.00	0.214	0.545	0.552	0.201
5	8.23	0.26	8.40	0.24		8.14	0.61	0.603	0.241	0.771	0.767
6	10.19	0.64	10.40	0.35		10.07	0.64	0.653	0.758	0.782	0.778
7	10.02	0.68	10.17	0.43		9.80	0.84	0.691	0.933	0.943	0.812

Total agreement (ICC)									0.781	0.825	0.717

**Table 7:** The volume assessment of the segmented teeth generated by CephX® and Insignia™.

Tooth no.	CephX		Insignia		ICC
	Mean	SD	Mean	SD	
*Maxilla*					
1	365.54	64.40	334.96	37.59	0.830
2	271.54	57.19	263.55	45.61	0.884
3	410.45	106.19	401.13	89.41	0.946
4	364.04	90.37	343.42	62.40	0.944
5	379.43	92.33	373.05	65.36	0.928
6	752.08	172.02	682.22	147.63	0.933
7	718.13	161.67	716.78	123.83	0.887

*Mandible*					
1	181.51	45.84	154.59	24.86	0.782
2	224.41	58.89	195.35	42.78	0.859
3	395.62	98.19	367.32	79.46	0.918
4	335.46	84.13	313.79	58.39	0.913
5	377.37	85.91	346.92	46.94	0.754
6	768.81	168.98	728.32	122.05	0.948
7	847.85	136.02	781.75	103.76	0.812

Total agreement (ICC)					0.881

## Data Availability

The data supporting the results of this study can be obtained from the corresponding author upon request.

## Additional Points

This study has several shortcomings, most significantly its small sample size. Customized orthodontic treatment is considerably more expensive than the conventional one; we could not obtain a larger sample of patients due to socioeconomic reasons. The other limitations were as follows: the clarity of CBCT images is affected by artefacts, noise, and poor soft tissue contrast; also, detecting accurate cross section during dimensional measurement with DICOM format may affect human errors.
